# Associating frailty and dynamic dysregulation between motor and cardiac autonomic systems

**DOI:** 10.3389/fragi.2024.1396636

**Published:** 2024-05-13

**Authors:** Patricio Arrué, Kaveh Laksari, Mark Russo, Tana La Placa, Meghan Smith, Nima Toosizadeh

**Affiliations:** ^1^ Department of Biomedical Engineering, University of Arizona, Tucson, AZ, United States; ^2^ Department of Aerospace and Mechanical Engineering, University of Arizona, Tucson, AZ, United States; ^3^ Department of Surgery, Division of Cardiac Surgery, Rutgers Robert Wood Johnson Medical School, New Brunswick, NJ, United States; ^4^ Arizona Center on Aging (ACOA), Department of Medicine, University of Arizona, Tucson, AZ, United States; ^5^ Division of Geriatrics, General Internal Medicine and Palliative Medicine, Department of Medicine, University of Arizona, Tucson, AZ, United States

**Keywords:** physiology network, convergent cross-mapping (CCM), frailty, cardiac autonomic system, motor performance, wearable sensors, heart rate dynamics, upper-extremity function

## Abstract

Frailty is a geriatric syndrome associated with the lack of physiological reserve and consequent adverse outcomes (therapy complications and death) in older adults. Recent research has shown associations between heart rate (HR) dynamics (HR changes during physical activity) with frailty. The goal of the present study was to determine the effect of frailty on the interconnection between motor and cardiac systems during a localized upper-extremity function (UEF) test. Fifty-six individuals aged 65 or above were recruited and performed the previously developed UEF test consisting of 20-s rapid elbow flexion with the right arm. Frailty was assessed using the Fried phenotype. Wearable gyroscopes and electrocardiography were used to measure motor function and HR dynamics. In this study, the interconnection between motor (angular displacement) and cardiac (HR) performance was assessed, using convergent cross-mapping (CCM). A significantly weaker interconnection was observed among pre-frail and frail participants compared to non-frail individuals (*p* < 0.01, effect size = 0.81 ± 0.08). Using logistic models, pre-frailty and frailty were identified with sensitivity and specificity of 82%–89%, using motor, HR dynamics, and interconnection parameters. Findings suggested a strong association between cardiac-motor interconnection and frailty. Adding CCM parameters in a multimodal model may provide a promising measure of frailty.

## Introduction

Frailty is an aging syndrome related to low physiological reserves in organs and systems and is associated with increased risk of hospitalization, adverse treatment outcomes, disability, and death (Fried et al., 2001). The primary indicators of frailty include muscle loss with normal muscle function, known as sarcopenia, and loss of muscle function without muscle mass loss, or dynapenia (Cruz-Jentoft et al., 2019; Kirk et al., 2024). These symptoms are triggered by metabolic and hormonal derangements (Serviddio et al., 2009; O’Connell et al., 2011; López-Armada et al., 2013; Johar et al., 2014), and the so called “heightened inflammatory state” (Yao et al., 2011), caused by excessive levels of C-reactive protein (CRP), proinflammatory cytokines interleukin 6 (IL-6), and white blood cells and tumor necrosis factor-alpha (TNFalpha) (Visser et al., 2002; Schaap et al., 2006; Yao et al., 2011). Consequently, frailty is highly associated with a decrease in motor function performance (Manini and Clark, 2012). Furthermore, frailty is associated with an impairment of the cardiac autonomic nervous system (ANS), given by alterations in the action potential on the sinoatrial node myocytes, which impacts the cardiac function and the heart rate variability (HRV) (Katayama et al., 2015). While research showed alterations in several physiological systems, such as in the endocrine, immune or digestive systems (Ghachem et al., 2021), the association between frailty and dynamic interconnection between cardiac and motor systems is still unclear. Indeed, the human body is a complex network of several physiological systems, where intricate dynamics exist between these systems to maintain homeostasis (Bashan et al., 2012; Bartsch et al., 2015). Accurate identification of the level of physiological reserve requires a collection of information across multiple physiological systems (Bashan et al., 2012; Bartsch et al., 2015; Ivanov et al., 2021; Romero-Ortuño et al., 2021), rather than only system-specific evaluations. To explore the extent of dynamic behaviors within and across physiological systems, principles of network physiology has been introduced. The concept of network physiology claims that dysregulation of interactions between physiological systems leads to loss of resilience and the ability to recover from stressors (Romero-Ortuño et al., 2021), which is inherent to the concept of frailty.

We have previously developed a methodology for assessing frailty that incorporates an upper-extremity function (UEF) and corresponding heart rate (HR) response to physical activity. The UEF test consists of repetitive and rapid elbow flexion and extension (Toosizadeh et al., 2017), during which several kinematics and kinetics features representing dynapenia are measured using motion sensors (Fried et al., 2001). Since UEF involves upper-extremity motion, it is feasible to perform for bedbound patients and where walking tests are difficult for frail older adults. In our recent research we showed that HR dynamics, measured by changes in HR due to the UEF physical function (i.e., HR dynamics), were significantly associated with frailty (Toosizadeh et al., 2021a). Combining UEF motor and cardiac functions, we were able to identify frailty with higher accuracy compared to models including each of the motor or HR dynamics parameters separately (Toosizadeh et al., 2022). Nevertheless, it is unclear whether frailty can influence the interconnection between motor and HR dynamics, and whether applying interconnection measures improve frailty identification.

The specific goal of the current study was to determine the effect of frailty on the interconnection between motor and cardiac systems. Build upon our previous research, the main hypothesis was that due to frailty, a weaker interconnection would exist between motor and HR performance. Recently, the concept of interconnection assessment within different physiological systems has gained attention (Heskamp et al., 2014; Heskamp et al., 2015; Schiecke et al., 2016; Verma et al., 2017; Schiecke et al., 2019; Schiecke et al., 2020). Granger causality is a classical approach that identifies causality between variables based on the removal of one to determine the predictability of the other variable (Granger, 1969), but its usage is limited to linear systems that have stationary behaviors, or for those in which variables are strongly coupled (Sugihara et al., 2012). In contrast, convergent cross-mapping (CCM) assesses the non-linear directional interactions of variables in a complex dynamic system, based on state-space reconstruction of time series collected from each system (Tsonis, 2018). The secondary hypothesis was that the accuracy of frailty identification would be improved using additional CCM interconnection parameters compared to models incorporating each of motor and HR parameters individually.

## Materials and methods

### Participants

Older adult participants (≥65 years) were recruited between October 2016 and March 2018. Participants were recruited from primary, secondary, and tertiary healthcare settings such as primary and community care providers, assisted living facilities, retirement homes, and aging service organizations. The inclusion criteria comprised individuals meeting the following conditions: 1) age 65 or above; 2) capable of walking a minimum distance of 4.57 m (15 feet) for frailty assessment; and 3) being able to understand and signing an informed consent form. The exclusion criteria involved: 1) severe motor disorders (such as Parkinson’s disease, multiple sclerosis, or recent stroke); 2) severe upper-extremity conditions (like bilateral elbow fractures or rheumatoid arthritis); 3) cognitive impairment assessed by a Mini Mental State Examination (MMSE) score of ≤23 (Folstein et al., 1975); and 4) terminal illness. Participants were also excluded if they had: 5) medical conditions or treatments that could disturb heart rate (HR) measurements, including arrhythmia or the use of a pacemaker; and 6) were taking β-blockers or similar medications that could impact HR, such as calcium or sodium channel blockers. Prior to participation, written informed consent was obtained from all individuals involved. The study was approved by the University of Arizona Institutional Review Board (approved IRB ID: 2105776487). All research was performed in accordance with the relevant guidelines and regulations, according to the principles expressed in the Declaration of Helsinki (World Medical Association, 2013).

### Frailty assessment and clinical measures

The assessment of frailty was performed using the established five-component Fried phenotype as the reference standard (Fried et al., 2001). The phenotype encompasses the following five criteria: 1) unintentional weight loss of 4.54 kg (10 pounds) or more within the previous year; 2) weakness in grip strength (adjusted with body mass index (BMI) and sex); 3) slowness measured by the required time to walk 4.57 m (15 feet) (adjusted with height and sex); 4) self-reported exhaustion gauged via a brief two-question version of the Center for Epidemiological Studies Depression (CES-D) scale; and 5) self-reported low energy expenditure based on a short version of the Minnesota Leisure Time Activity Questionnaire (Fieo et al., 2013). Participants were classified into three frailty categories: non-frail if they met none of the criteria, pre-frail if they met one or two criteria, and frail if they met three or more criteria. Several clinical measures were collected, including: 1) assessments of cognition using the Mini Mental State Examination (MMSE) and Montreal Cognitive Assessment (MoCA) (Folstein et al., 1975; Nasreddine et al., 2005); 2) determination of comorbidities using the Charlson Comorbidity Score (CCI) (Charlson et al., 1987); and 3) assessment of depression through the Patient Health Questionnaire (PHQ-9) (Kroenke et al., 2001). These measures were included as adjustment variables in the statistical analysis, considering their potential influence on both physical activity and cardiovascular system performance.

### Combined motor and cardiac functions assessment during UEF test

After completing the frailty assessment and clinical measures, participants were instructed to sit on a chair and rest for 2 minutes to regain a normal resting state. Following this, they performed the UEF task of elbow flexion-extension as rapidly as possible for 20 s using their right arm. Subsequently, participants rested on the chair for an additional 2 minutes. Notably, our previous research demonstrated comparable UEF outcomes for both the left and right arms (Toosizadeh et al., 2015). Before the test session, participants familiarized themselves with the UEF protocol by practicing with their non-dominant arm. Clear instructions were given using exact verbal cues, encouraging participants to perform the task as fast as possible, just before initiating the elbow flexion task. Wearable motion sensors, specifically triaxial gyroscope sensors (BioSensics LLC, Cambridge, MA) operating at a sampling frequency of 100 Hz (Supplementary Figure S1A, on Supplementary Material), were employed to capture forearm and upper arm motions, and eventually the elbow angular velocity. The angular velocity data obtained from the gyroscopes underwent filtering using a first-order high-pass Butterworth filter set at a cutoff of 2.5 Hz. The maximums and minimums of the angular velocity signal were identified to determine the cycles of elbow flexion. The assessment of motor performance involved multiple aspects: 1) slowness measured by elbow flexion speed; 2) flexibility assessed by range of motion; 3) weakness evaluated based on upper-extremity muscle strength; 4) variability in speed and motor accuracy; 5) fatigue determined by the decline in speed during the 20-second task; and 6) the number of flexion cycles. The normalized UEF motor score, ranging from zero (indicating resiliency) to one (indicating extreme frailty), was calculated by summing the sub-scores corresponding to performance outcomes and demographic data (i.e., BMI) (Toosizadeh et al., 2017). The normalized UEF motor score, ranging from zero (indicating resiliency) to one (indicating extreme frailty), was calculated by summing the sub-scores corresponding to performance outcomes and demographic data (i.e., BMI) (Toosizadeh et al., 2017). More detailed information about UEF validation, repeatability, and the normalized score can be found in prior research (Toosizadeh et al., 2015; Toosizadeh et al., 2016; Toosizadeh et al., 2017).

Heart rate was recorded using a wearable ECG device equipped with two electrodes and a built-in accelerometer (360° eMotion Faros, Mega Electronics, Kuopio, Finland; ECG sampling frequency = 1,000 Hz, accelerometer sampling frequency = 100 Hz; Supplementary Figure S1A). One ECG electrode was positioned on the upper mid-thorax, while the other was placed inferior to the left rib cage. This electrode configuration minimized movement artifacts during the UEF test with the right arm. The recorded ECG data underwent analysis, encompassing a 20-second baseline, 20-second UEF task, and a 30-second recovery period. RR intervals (successive R peaks of the QRS signal) were computed using the Pan-Tompkins algorithm (Pan and Tompkins, 1985). The automated peak detection process was manually reviewed by two researchers (PA and NT). Previously, two categories of HR parameters were derived: one representing baseline HR and HRV (such as RMSSD (Shaffer and Ginsberg, 2017)), and the other reflecting HR dynamics (changes in HR during UEF and HR recovery after the task) (Toosizadeh et al., 2022). We computed RMSSD based on ultra-short HRV parameters during rest (20-seconds) (Arêas et al., 2018; Shaffer et al., 2020). The HR dynamics parameters included the time taken to reach maximum and minimum HR, as well as the percentage increase and decrease in HR during activity and recovery periods, respectively. In addition to previously developed parameters, in the current study, the interconnection between motor and HR data were assessed using CCM.

### CCM analysis

We quantitatively assessed the directional nonlinear interactions between HR and motor data using CCM. An overview of the method is summarized in Supplementary Figure S1. CCM tests whether a historical trace of HR can predict motor performance (or inversely, whether a historical trace motor performance can predict HR). To calculate the CCM, we first created evenly sampled data of synchronized HR and motor function with a sampling frequency of 10Hz, using spline interpolation (Supplementary Figure S1B). Each HR data point represents average HR values over 0.1 s. Corresponding motor data represent the angular displacement travelled during each 0.1 s of UEF. For calculating motor performance, motor function $M_{f}$ was defined in Eq. 1,

$${M_{f}}_{i}=\int_{t_{i}}^{t_{i}+0.1}\omega_{e}dt,$$ (1)

where $\omega_{e}$ represents the rectified angular velocity of the elbow.

Taken’s embedding theorem generally guarantees that the space state of a dynamic system could be represented from a single-observed time series X as an E -dimensional manifold (Takens, 1981). The shadow or reconstructed manifold, denoted by $M_{X}$ , consists of an E -dimensional data with lagged coordinates ( τ ) of the variable as shown in Eq. 2:

$$M_{X}=\langle X\left(t\right),X\left(t-\tau \right),X\left(t-2\tau \right)\ldots X\left(t-\left(E-1\right)\tau \right)\rangle .$$ (2)

Subsequently, we reconstructed E -dimensional manifolds from each of these two time series (Takens, 1981) (Supplementary Figure S1C). Dimension ( E ) of four was selected based on the average false nearest neighbor approach (Cao, 1997). A time lag ( τ ) of 1 s was used for analysis based on the delayed mutual information method applied to ECG and motor data time series (Leng et al., 2020). We predicted one time series (e.g., motor function) by historical records of the other signal (e.g., shadow manifold of HR) using a k-nearest neighbor technique. For a dimension E , we determined E+1 nearest neighbors and identified indices of each data points in manifolds ( $M_{X}$ ). Using these indices for one manifold (e.g., motor data $X\left(t\right)$ ), we found corresponding neighbors in the second manifold (e.g., HR data $Y\left(t\right)$ ) (Supplementary Figure S1D), and then predicted $X\left(t\right)$ to $\hat{Y}\left(t\right)$ as the weighted mean of E+1 points in the second manifold shown in Eq. 3 (Tsonis et al., 2018):

$$\hat{Y}\left(t\right)=\sum_{i=1}^{E+1}w_{i}Y\left(t_{i}\right),$$ (3)

where $w_{i}$ weights are calculated based on the Euclidean distances between $M_{Y}$ and its $i^{th}$ nearest neighbor on $X\left(t\right)$ .

In the literature, CCM interconnection strength is quantified by the Pearson correlation coefficient between the predicted and original time series (Supplementary Figure S1E). We also compute the normalized root-mean-square-error (NRMSE) as a complementary indicator to determine magnitude of difference in predicted vs. actual time series (rather than the shape of differences measured by Pearson correlation coefficient). NRMSE was calculated by normalizing the RMSE between the predicted and the ground truth with respect to the standard deviation of observations. As documented in previous studies, the correlation coefficient is expected to increase with increasing the time-series length (i.e., library length, Supplementary Figure S1F). For the current study, the correlation and NRMSE values were calculated at the maximum library length (Supplementary Figure S1F).

### Statistical analysis

Shapiro-Wilk W test was applied to demographics to test for normality. Student t-test models were employed to assess normal demographic differences across frailty groups, excluding sex, which was analyzed using the chi-square (χ^2^) test to evaluate differences in sex distribution among frailty groups, and MMSE, MoCA, CCI and PHQ-9 questionnaires, for which, differences were analyzed through Wilcoxon rank sum test for not normally distributed data. When comparing CCM parameters among frailty groups, multivariable ANOVA models were utilized, adjusting for age, sex, and BMI due to their previously established associations with motor performance, cardiac function, and frailty (Hubbard et al., 2010; Gordon et al., 2017; Toosizadeh et al., 2017; Anderson et al., 2018). Cohen’s effect size (*d*) was calculated to estimate the magnitude of differences. Further analyses involved repeating ANOVA comparisons for CCM parameters across frailty groups, incorporating clinical measures significantly associated with frailty as covariates. To evaluate the additional value of interconnection measures compared to prior models utilizing individual motor and HR parameters, logistic regression models were constructed. These models had frailty as the dependent variable, while HR, motor, and CCM parameters served as independent variables. A stepwise parameter selection, based on Akaike information criterion (AIC) values, was applied to identify independent predictive variables. Receiver operating characteristic (ROC) curves were generated to compute the area under the curve (AUC) with a 95% confidence interval (CI) for each predictive model. All statistical analyses were conducted using JMP (Version 16, SAS Institute Inc., Cary, NC, United States of America), and significance was determined at *p* < 0.05.

## Results

### Participants and clinical measures

The study included 56 participants, comprising 12 non-frail individuals (age = 76.92 ± 7.32 years), 40 pre-frail individuals (age = 80.53 ± 8.12 years), and four frail individuals (age = 88.25 ± 4.43 years). Due to the limited number of participants in the frail group, the frail and pre-frail groups were combined for the statistical analysis. A summary of demographics is provided in Table 1. Importantly, no significant difference was observed in demographic parameters among the frailty groups (*p* > 0.10). However, among clinical measures, there were significant differences in CCI comorbidity and PHQ-9 depression scores between the frailty groups (*p* < 0.03, Table 1).

**TABLE 1:** Demographic information and clinical measures of participants. Student t-test, Chi-square (for sex) and Wilcoxon rank sum test were used to study differences, post Shapiro-Wilk normality test.

Variables	Non-frail (n = 12)	Pre-frail/Frail (n = 44)	*p-value (effect size)*
Female, n (% of the group)	7 (58%)	34 (77%)	0.10
Age, year (SD)	76.92 (7.32)	81.23 (8.14)	0.05 (0.54)
Height, cm (SD)	164.36 (9.13)	164.23 (10.27)	0.12 (0.01)
Weight, kg (SD)	66.58 (14.69)	75.53 (19.56)	0.07 (0.48)
Body mass index, kg/m^2^ (SD)	24.67 (5.55)	27.74 (5.71)	0.10 (0.54)
MMSE score, 0–30 (SD)	29.67 (0.65)	29.14 (1.34)	0.15 (0.19)
MoCA score, 0–30 (SD)	26.25 (3.08)	24.88 (2.80)	0.06 (0.25)
CCI score, 0–29 (SD)	1.42 (1.78)	3.86 (2.89)	<0.01* (0.37)
PHQ-9 score, 0–30 (SD)	0.42 (0.51)	2.35 (2.89)	<0.01* (0.35)
Fried criteria, n (% of the group)			
Weight loss	0	1 (2%)	
Weakness	0	18 (41%)	
Slowness	0	34 (77%)	
Exhaustion	0	7 (16%)	
Low energy	0	8 (18%)	

### CCM analysis

Significant effects of frailty on CCM correlation values were observed for interconnections in both directions, including predicting HR time series based on motor function (motor to HR) and predicting motor function based on HR (HR to motor) as reported in Table 2, Supplementary Figures S2 and S3 (see Supplementary Material). Pre-frail/frail older adults showed smaller correlations in CCM for both directions, compared to non-frail older adults (*p* < 0.03). There was also a significant effect of frailty on NRMSE values; for both motor and HR CCM predictions, NRMSE values were significantly smaller among non-frail compared to pre-frail/frail (*p* < 0.04).

**TABLE 2:** Differences in UEF, HRV, and CCM features across frailty groups. ANOVA was used to study differences. A significant association is represented by the asterisk.

Parameters	Non-frail (N = 12)	Pre-frail/frail (N = 44)	*p*-value (effect size)
UEF MOTOR SCORE^a^			
UEF MOTOR SCORE, 0–1 (SD)	0.32 (0.18)	0.53 (0.23)	0.01* (0.90)
HR DYNAMICS PARAMETERS^a^			
HR MEAN, BASELINE, BPM (SD)	71.52 (11.38)	77.59 (15.97)	0.23 (0.43)
RMSSD, BASELINE, MS (SD)	16.80 (18.14)	14.76 (16.20)	0.74 (0.12)
HR PERCENT INCREASE, % (SD)	19.28 (7.55)	10.29 (4.79)	<0.01*(1.49)
HR PERCENT DECREASE, % (SD)	15.24 (7.65)	8.13 (3.97)	<0.01*(1.25)
CCM PARAMETERS			
CORRELATION MOTOR→HR (SD)	0.81 (0.10)	0.69 (0.21)	0.03*(0.77)
CORRELATION HR→MOTOR (SD)	0.72 (0.19)	0.53 (0.26)	0.01*(0.89)
NRMSE MOTOR→HR, % (SD)	57.28 (14.47)	66.10 (13.26)	0.02*(0.86)
NRMSE HR→MOTOR, % (SD)	65.82 (21.67)	81.68 (19.00)	0.04*(0.73)

UEF, upper extremity function; SD, standard deviation; HR, heart rate; bpm: beats per minute; CCM, convergent cross-mapping; NRMSE, normalized root mean square error.

^a^ : results of a previous study (Toosizadeh et al., 2022).

**FIGURE 1:**
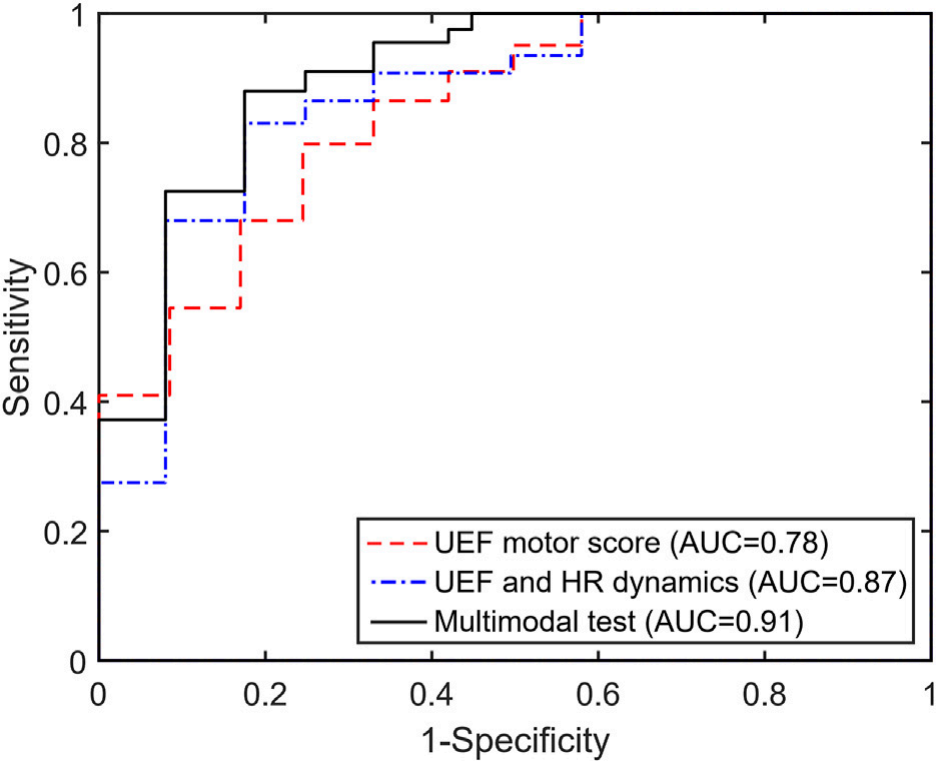
The area under the receiver operator characteristics (ROC) curve for the UEF motor score, the previous multimodal test (HR + UEF motor score), and the current model incorporating the CCM parameter (HR + UEF motor + CCM).

Within the stepwise regression analysis, UEF score, HR percent increase, and CCM Motor-to-HR parameters were selected as independent predictors of frailty categories (non-frail vs. pre-frail/frail). Using these three parameters, pre-frailty/frailty was predicted with an AUC, sensitivity, and specificity of 0.91, 0.89, and 0.83 (Table 3; Figure 1), which had a 7% higher AUC than models that included only individual motor or HR parameters as predictors.

**TABLE 3:** Logistic models for predicting frailty using CCM parameters, compared to UEF and HR dynamics models developed in previous research. A significant association is represented by the asterisk.

Independent variable	Parameter estimate	Standard error	Chi-square ( $\chi^{2}$ )	*p*-value (95% CI)
Model 1–UEF motor score (AUC = 0.78; AICc = 53.94; Sensitivity = 0.75; Specificity = 0.75)				
Intercept	0.61	0.73	0.70	0.4 (−0.81:2.12)
UEF motor score	−0.05	0.02	6.85	<0.01 (−0.08:-0.01)*
Model 2–HR dynamics (AUC = 0.84; AICc = 44.25; Sensitivity = 0.80; Specificity = 0.75)				
Intercept	−4.91	1.27	14.97	<0.001 (-7.92: 2.81)*
HR percent increase	0.25	0.08	10.38	<0.001 (0.12:0.44)*
Model 3–CCM (AUC = 0.74; AICc = 55.60; Sensitivity = 0.75; Specificity = 0.25)				
Intercept	4.21	1.52	7.72	<0.01 (1.77:7.86)*
CCM HR→M	−4.53	2.11	4.59	0.03 (−9.45: 0.98)*
Model 4–Combined UEF and HR dynamics (AUC = 0.87; AICc = 76.67; Sensitivity = 0.82; Specificity = 0.83)				
Intercept	−3.21	1.55	4.28	0.04 (−6.68: 0.45)*
HR percent increase	0.23	0.08	7.73	<0.01 (0.09:0.42)*
UEF motor score	−0.03	0.02	2.67	0.10 (−0.07:0.01)
Model 5–Combined UEF, HR dynamics and CCM (AUC = 0.91; AICc = 42.80; Sensitivity = 0.89; Specificity = 0.83)				
Intercept	6.26	2.83	4.88	0.03 (1.69:13.14)*
HR percent increase	0.22	0.08	7.38	<0.01 (−0.42: 0.08)*
UEF motor score	−0.03	0.02	2.69	0.10 (−0.00:0.08)
CCM HR→M	4.54	2.90	2.45	0.12 (−11.27:0.51)

HR, heart rate; UEF, upper-extremity function; CCM, convergent cross-mapping; AUC, area under curve; CI, confidence interval; AICc, Akaike’s information criterion with correction for small sample size.

## Discussion

### Effect of frailty on system interconnections

As hypothesized, a significantly weaker interconnection between motor and cardiac systems was observed among pre-frail and frail older adults compared to non-frail individuals (Table 2; Supplementary Figure S2). Indeed, these results are consistent with expected changes due to aging-related physiological dysregulation. Autonomic nervous system (ANS) regulates heart activity during exercise using signals from the central nervous system (Williamson et al., 2006) and feedback mechanisms from the exercise pressor reflex (group III and IV muscle afferents) (Kaufman and Hayes, 2002) and the arterial baroreflex, which controls blood pressure and consequently cardiac output (Charkoudian et al., 2005). Previous studies have shown that exercise pressor reflex is impacted by aging (Caron et al., 2018; Trinity et al., 2018; Hasegawa et al., 2021; Teixeira and Vianna, 2022), which would potentially alter the interconnection between the motor and cardiac systems. Nevertheless, the effect is still controversial and further research is needed to fully understand this interconnection pathway, which, in the current study, was quantified through CCM parameters. One potential explanation is that frailty leads to an altered control of motor to cardiac system by affecting exercise pressor reflex. Nevertheless, this hypothesis should be investigated in future research.

In addition to exercise pressor reflex, the observed weaker CCM values among pre-frail and frail older adults may be explained by the general concepts of homeostatic physiological dysregulation and heightened inflammatory state (Yao et al., 2011; Milot et al., 2014). In this regard, aging and more specifically frailty can be caused by breakdowns of key regulatory processes and excessive increase of immune factors, leading to the loss of homeostasis and functional impairment (Visser et al., 2002; Serviddio et al., 2009; O’Connell et al., 2011; Yao et al., 2011; López-Armada et al., 2013). Different methods have been used previously to identify physiological dysregulation, such as Mahalanobis multivariate statistical distance and principal component analysis. Mahalanobis multivariate statistical distance is a multivariate model built to assess dysregulation within relevant blood-based biomarkers for frailty, such as red blood cell count, IL-6, CRP, calcium, and hemoglobin (Cohen et al., 2013). This method showed that the increase in the multivariate distance is accelerated with age, which represents the loss of integration of the system physiology. Similarly, the principal component analysis approach considered the variability of blood-based biomarkers, and consequently was showed to be an independent frailty predictor (Nakazato et al., 2020). Both methods included information from multiple systems to assess frailty, analogously to how CCM parameters were computed from HR and motor time-series. CCM results support the fact that interconnection measures from motor and heart systems are strongly associated with frailty.

### Frailty identification using multimodal models

The present study builds upon prior research by integrating a methodology previously developed for the concurrent assessment of both motor and cardiac autonomic control systems. In addition to this established framework, we incorporated the assessment of the dynamic interaction between these systems. Current findings confirmed that assessing these two physiological systems and their interaction can improve frailty identification compared to models that focus on data from individual physiological systems. Cardiac and motor systems were selected in this study as they are strongly associated with frailty. Muscle loss and weakness stand as primary indicators of frailty, often stem from inflammatory, metabolic, and hormonal derangements (Visser et al., 2002; Schaap et al., 2006; Serviddio et al., 2009; O’Connell et al., 2011; Yao et al., 2011; Clark and Manini, 2012; Manini and Clark, 2012; López-Armada et al., 2013; Johar et al., 2014). Motor deficits and muscle weakness are commonly assessed using walking speed or grip strength tests (Fried phenotype) or counting deficits/disorders (Rockwood deficit index) (Rockwood et al., 2005; Rockwood and Mitnitski, 2007). Nevertheless, performing walking tests in the clinical setting is cumbersome, and many frail older adults have walking disabilities. Grip strength, on the other hand, only measures muscle strength and cannot reveal other aspects of motor deficits. We have previously validated the sensor based UEF motor task to accurately recognize consistent declines in motor performance associated to frailty, including diminished speed, weakness, rigidity, fatigue, and motor variability (Toosizadeh et al., 2016; Ehsani et al., 2019).

In addition to the motor system, the implemented method included cardiac autonomic control. Prior studies have highlighted an association between frailty and an impaired autonomic nervous system (ANS) marked by alterations in electrical conduction and action potential structure (Varadhan et al., 2009; Moghtadaei et al., 2016). The compromised neurohormonal balance associated to ANS dysfunction is correlated with health complications (Arslan et al., 2008; Zebrowski et al., 2015). HRV, specifically the variability in RR intervals within QRS-waves during rest, has been utilized to assess ANS dysfunction and proposed as a critical physiological marker (Kleiger et al., 1987; Muller et al., 1989; Morris and Norris, 2005). Nonetheless, variations in resting HRV exist among individuals and throughout the day, influenced by factors such as breathing patterns and environmental conditions (Taelman et al., 2008; Valencia et al., 2008; Cipryan and Litschmannova, 2013). In the present research, we showed an additional advantage of collecting HR during UEF activity, as we could directly assess cardiac physiological reserve in response to a controlled stressor (UEF physical task), establishing a stress-response model that was further used for assessing interconnection measures between motor and HR.

As the last component, within the current study, we investigated the interconnection between physiological systems in response to stress caused by the physical task. The concept of stress-response testing for quantifying frailty has become the subject of recent research. Evidence suggests that differences in physiological reserve between non-frails and frails are subtle under the basal condition (Varadhan et al., 2008). Implementing provocative testing accentuates frailty-related alterations in measurable dynamics of physiological systems in response to stimuli. The provocative UEF test is designed to be hard enough to stress motor and cardiac systems, and not too demanding, so they can be incorporated in a routine clinical setting for frail older adults, especially those with walking disabilities. Simultaneous assessment of motor and heart function in this manner allows us to accurately quantify the dysregulation of interconnection between these systems. Further, the motion artifacts are minimum with the proposed testing, with HR measurement acquiring from the left side while the participant perform the physical task on the right side.

### Limitations and further work

Despite the promising findings of the current study, there are some limitations related to the recruited sample. First, the sample size of community-dwelling older adults chosen for this research was small. Second, the number of frail participants was limited, leading to the combination of pre-frail and frail groups for analysis purposes. Third, individuals with arrhythmia and those needing β-blockers or pacemakers were excluded from participation in the study. Also, test-retest reliability of CCM parameters were not investigated here. Therefore, the interconnection analysis should be confirmed in larger studies incorporating test-retest reliability measures. Additionally, we used time-series library lengths that may not provide accurate results for a few participants, since some HR data may have a higher level of short-term complexity, leading to less dense attractor shadow manifolds and consequently a non-completely developed convergence of CCM parameters. Possible solutions would be to perform longer arm tests; however, this would lead to more physical demand on frail older adults. In addition, Recent findings indicate that ultra-short (∼20-second) HRV measurements demonstrate no significant differences compared to the outcomes derived from short-term (5-min) evaluations. However, comprehensive validation is still required for these ultra-short measurements (Arêas et al., 2018; Shaffer et al., 2020). In addition, due to the short period of HR data collection, we were unable to calculate certain HRV parameters such as entropy measures. Lastly, approximately 10% of phenotypically healthy older adult participants may have had latent parkinsonism (Gibb and Lees, 1988); however, they may not have been excluded from the present study. In future research, we would consider the addition of the UPDRS-III clinimetric scale (Kremer et al., 2023) because it is similar to the UEF test and also may assist in identifying Parkinson’s disease, to assure our exclusion criteria is correctly applied. Further, we have previously developed a dual-task module for assessing cognitive impairment using the UEF test (Toosizadeh et al., 2021b; Eskandari et al., 2022; Ruberto et al., 2022; Toosizadeh et al., 2022). In our future work, we will use this module to understand the three-way interconnection between brain function, motor performance, and HR, using functional near-infrared spectroscopy (fNIRS) for measuring brain function time series.

## Conclusion and clinical implications

In the present work a novel quantification of interconnection between motor and cardiac autonomic systems was implemented for frailty assessment. We demonstrated that CCM parameters showed weaker interconnection between motor and cardiac systems among pre-frail/frail older adults compared to non-frails. The new CCM parameters also showed promising results in improving frailty prediction within logistic models. The simplicity of the investigated UEF test permits performing it even for hospitalized bed-bound patients, for predicting therapy complications, in-hospital outcomes, and rehabilitation strategies. We expect to present this multimodal test as an alternative to accurate but impractical frailty assessment tools such the Fried phenotype, when patients are not able to walk. Further, commercialized wearable devices are now allow accurate assessment of HR and motion. Showing the proof of concept in the current study, in our future investigation, we will develop an easy-to-use app for Smart Watch for identifying frailty using simultaneous measures of motor and cardiac functions.

## Data Availability

The raw data supporting the conclusion of this article will be made available by the authors, without undue reservation.
