# Supplementary figures and images for: Associating frailty and dynamic dysregulation between motor and cardiac autonomic systems
Source: Front Aging. 2024 May 13;5:1396636. doi: 10.3389/fragi.2024.1396636 (PMC11128670; doi:10.3389/fragi.2024.1396636)

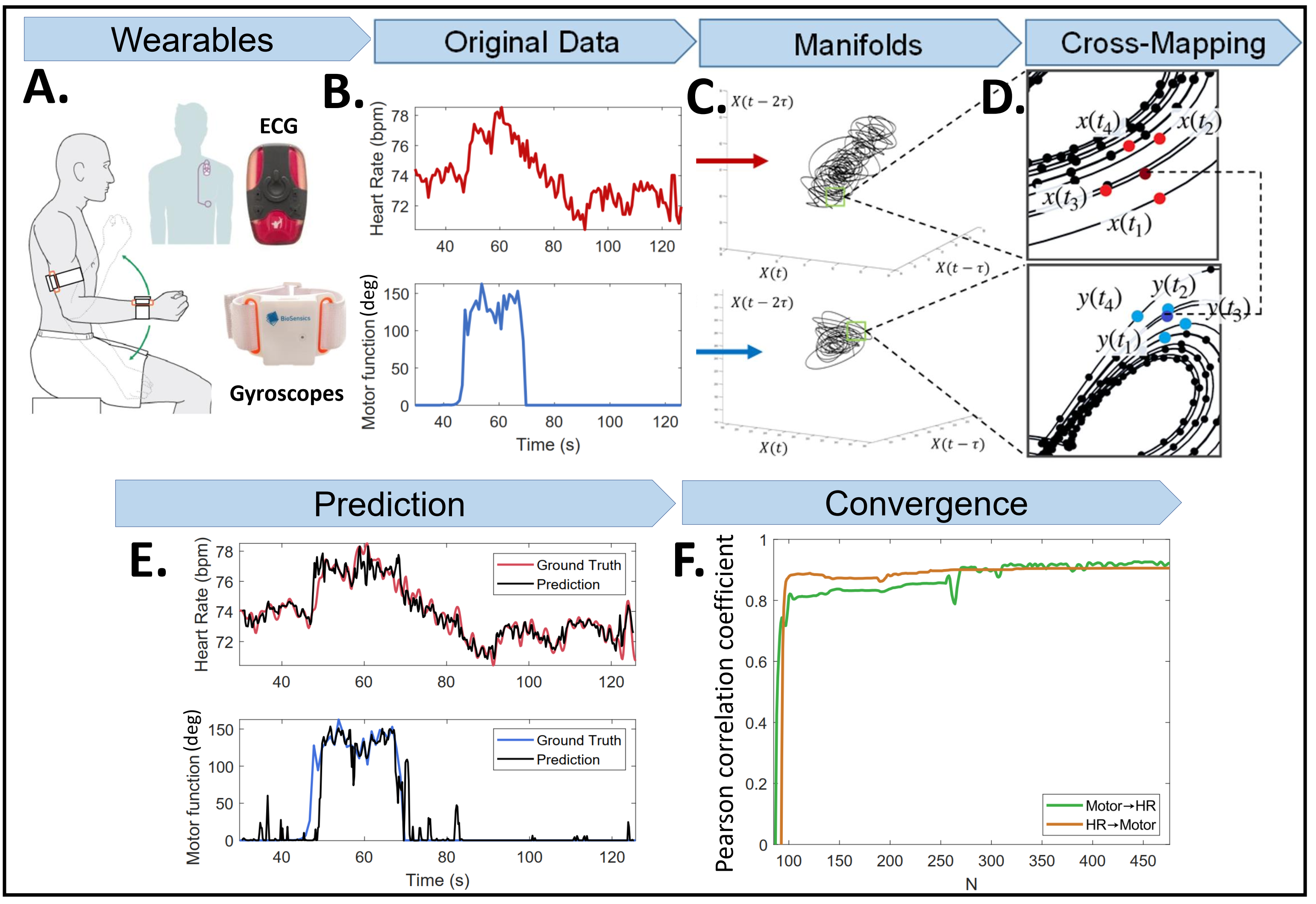

Supplement: Supplementary file 1 [file Image3.TIF]

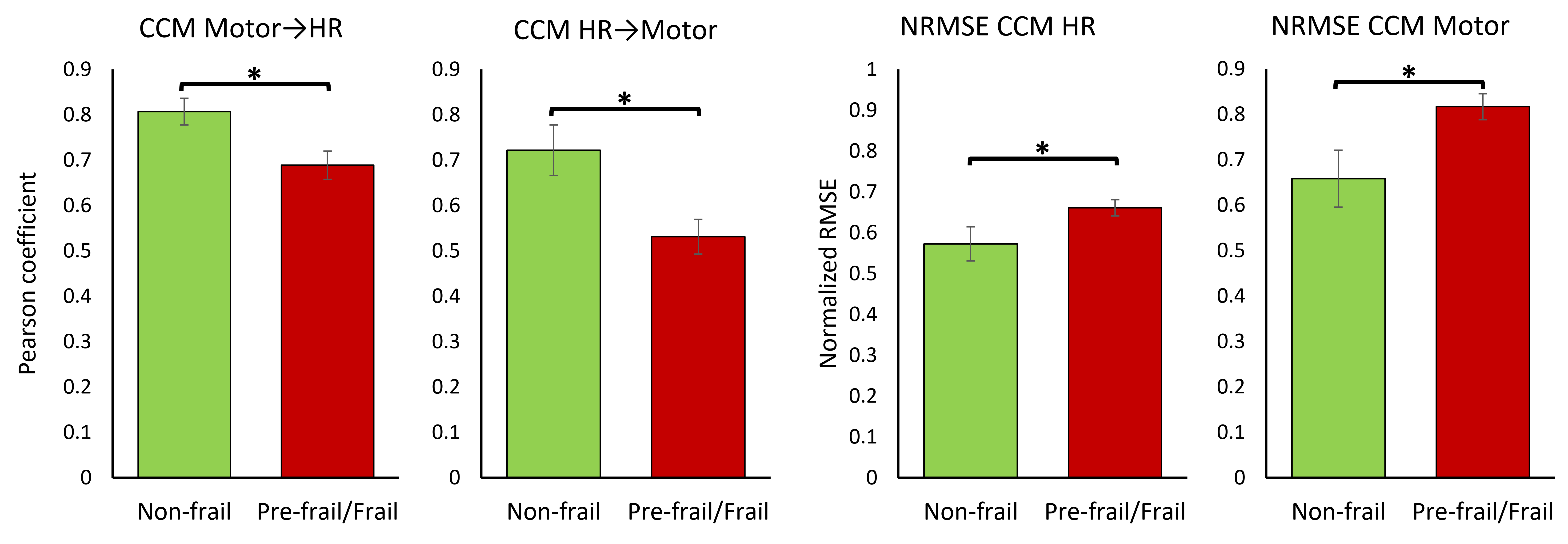

Supplement: Supplementary file 2 [file Image2.TIF]

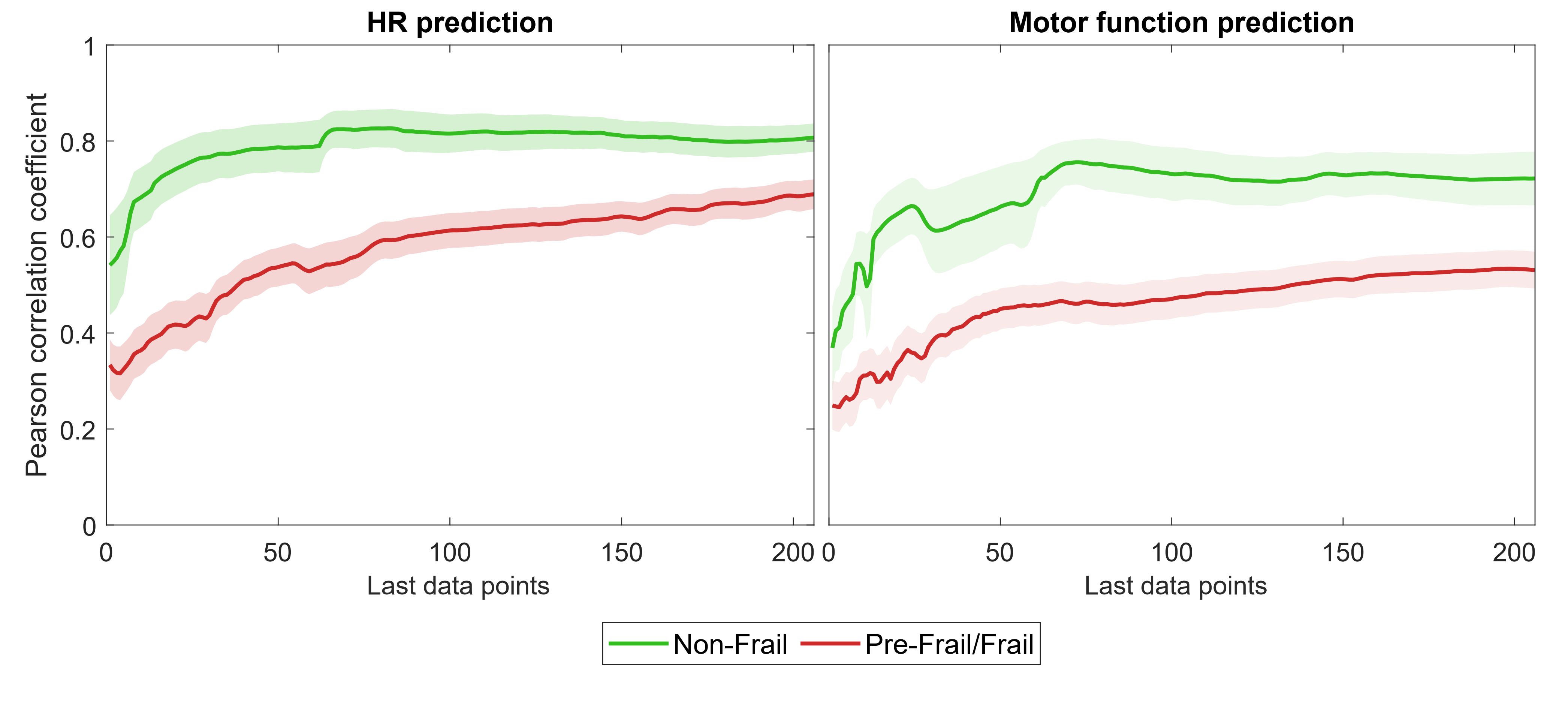

Supplement: Supplementary file 3 [file Image1.TIF]
