# Supplementary material for: Associating frailty and dynamic dysregulation between motor and cardiac autonomic systems
Source: Front Aging. 2024 May 13;5:1396636. doi: 10.3389/fragi.2024.1396636 (PMC11128670; doi:10.3389/fragi.2024.1396636)
Supplement: Supplementary file 4 [file DataSheet1.PDF]

Supplementary materials

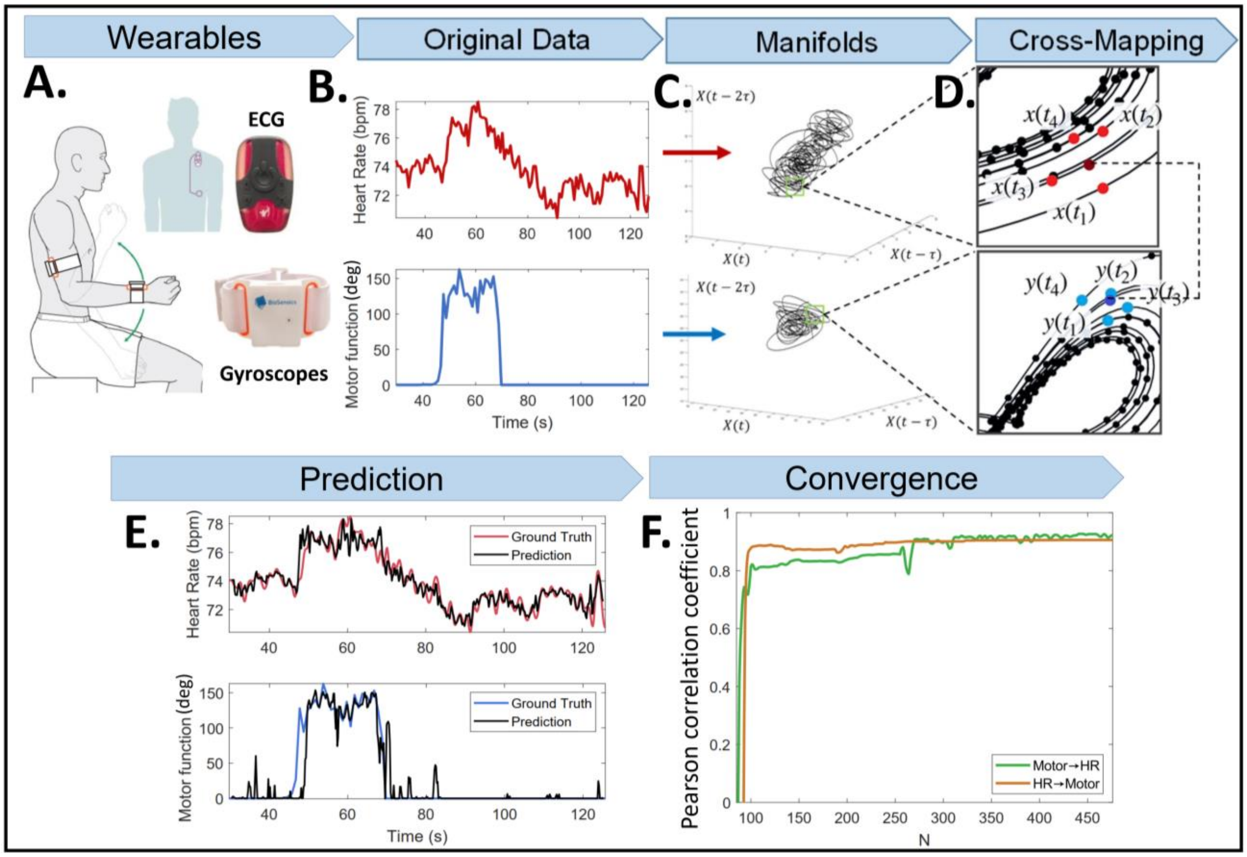

Figure S1: Overview of the CCM method to assess interconnection between motor and HR data: A) Wearable devices (gyroscopes) to obtain angular velocity and ECG during the UEF physical task; B) Motor performance and HR extraction; C) CCM shadow attractor manifolds on time-lagged coordinate systems; D) Prediction of HR from motor function and vice-versa in a time point (dark red and dark blue dots, respectively) using a distance-based weighted average of neighbors (bright red and bright blue dots); E) Comparison between predicted motor (or HR) data and ground truth; and F) Convergence curves of Pearson correlation coefficient between predicted and ground truth as a function of library length (data points used for developing manifolds).

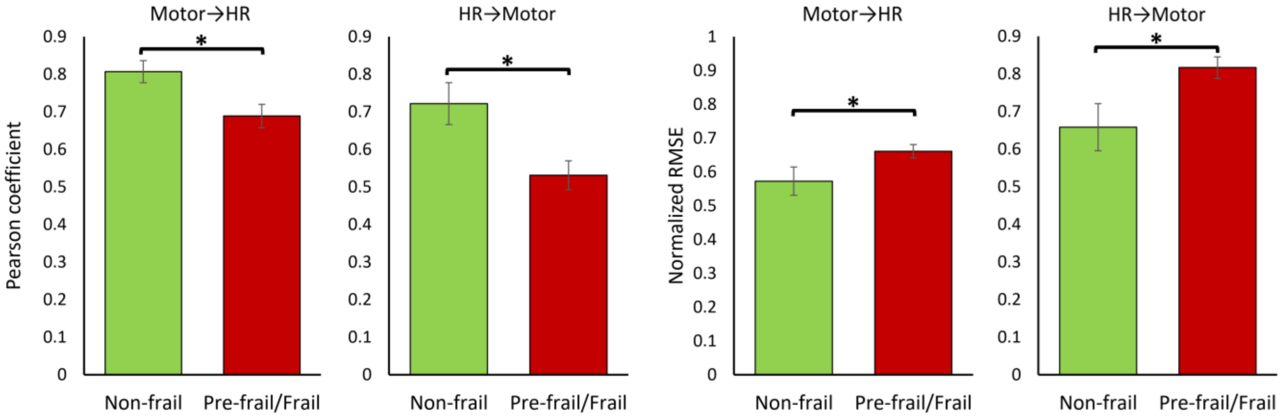

Figure S2. CCM parameters and NRMSE across frailty groups. A significance between group difference is identified by the asterisk (p<0.05).

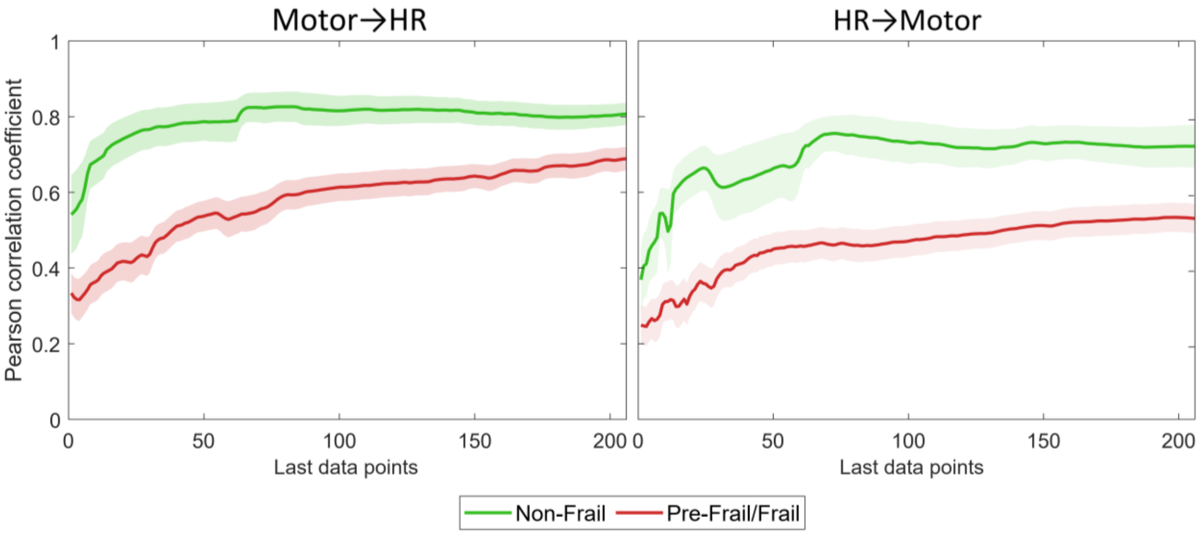

Figure S3. Convergence curves distribution for CCM predictions. Solid lines represent the average across each group at each library length and shaded regions show the standard error.
